# Conceptual Models of Social Determinants of Health: A Narrative Review

**Published:** 2017-04

**Authors:** Sayyed Morteza HOSSEINI SHOKOUH, Mohammad ARAB, Sara EMAMGHOLIPOUR, Arash RASHIDIAN, Ali MONTAZERI, Rouhollah ZABOLI

**Affiliations:** 1. Dept. of Health Services Management, Faculty of Health, Baqiyatallah University of Medical Sciences, Tehran, Iran; 2. Dept. of Health Management and Economics, School of Public Health, Tehran University of Medical Sciences, Tehran, Iran; 3. Mental Health Research Group, Health Metrics Research Center, Institute for Health Sciences Research, ACECR, Tehran, Iran

**Keywords:** Conceptual model, Social determinants of health, Health inequalities, SES indicator

## Abstract

**Background:**

There are several conflicting conceptual models to explain social determinants of health (SDH) as responsible for most health inequalities. This study aimed to present these models in historical perspective and provide main component of SDH models as an SES indicators.

**Methods:**

This was a narrative study using international databases to retrieve literature dealing with conceptual models of SDH. All publication in English language until Mar 2015 was included. The CASP and PRISMA were used to summarize the literature.

**Results:**

Overall, 248 publications were retrieved and screened. After exclusion of irrelevant and duplicates, 94 citations were found to be relevant and 21 publications included in this review. In general, 21 models of SDH were found: some models presented before year 1995(n=4), some models presented between 1995 and 2005 (n=13) and some models presented after 2005 (n=4). However, we found three categories of indicators that contribute to SDH models and that were classic factors, fixed and demographic factors and proxy factors.

**Conclusion:**

Reduction of socioeconomic inequalities in health requires understanding of mechanisms and causal pathways; therefore, every country needs to design the specific model. As the available models are for developed countries, lack of a specific model for developing ones is tangible. As there is no gold standard related to SES indicators, therefore, it is proposed to use the various indicators based on life course approach, which leads to understanding and adopting effective policy interventions.

## Introduction

At present, “good health is an international acceptable goal” for people and communities. Thus, in the last century, great successes were achieved in some health indicators such as life expectancy although health inequalities still exist between rich and poor (1).

Health is a multidimensional issue that various factors are influencing on its supply, development or destruction. All people, systems, and organizations in the society play a role in making and receiving the health outcomes (2–4). Among the factors which affecting health, the share of healthcare, biological factors, physical, environmental and behavioral factors and socioeconomic determinants are 25%, 15%, 10% and 50%, respectively (5).

Currently, the most fundamental causes of health inequalities are related to different socioeconomic conditions (5), also the most serious factors of illness are related to socio-economic conditions in which people work and live (6). This condition in the literature is known as “the causes of the causes” (7). Although an effective good healthcare is necessary for community health improvements but never enough for encountering the health challenges and overcoming the health inequalities, which SES plays significant role and large part of health problems is related to them (8,9). Due to importance of socioeconomic determinants of health, the social determinants of health (SDH/SDoH) are used at health literature by WHO and established the Commission on Social Determinants of Health (CSDH) in 2005 (7, 10). These determinants referring to the conditions in which people born, grow up, live, work, age and inequalities in this cases lead to health inequalities (7, 9–12).

In recent years, international trends show a significant increase in measuring and documenting the social determinants of health inequalities for health policy (13) and this determinant are known as the most obvious cause of inequalities in health of countries (8, 10–12, 14). In the literature of social epidemiology and health economics, SES is identified based on three main traditional classics indicators: income, education, and occupation (15, 16). however, another variables such as: hosing (17, 18), smoking (18–20), BMI (18–21), access to health care services (22), ethnicity (17, 23), insurance coverage (24), residence area (18), religious (16–18, 25), physical activity (19), and social capital (17) also has been emphasized and measured.

Since better understanding of health inequalities requires recognizing the main causes, direct and indirect effects mechanism of them, the conceptual models of socioeconomic status (SES) or social determinants are used. As different models were presented to describe public health (26–37), these models are also used and shaped different conceptual models of SDH. WHO, at the international level, has emphasized the importance of SDH to assess the health inequalities in middle- and low-income countries (25, 38, 39) and with regards to different pathways, mechanisms and indicators suggested by different and conflict conceptual models of SDH, motivated us to present these models in historical perspective and provide main component of SDH models as an SES indicators.

## Materials and Methods

### Definitions

#### 1- Conceptual model

The conceptual model is the system of concepts, assumptions, expectations, beliefs, and theories that support and informs research framework. A conceptual model was defined as a visual or written product, one that “explains, either graphically or in narrative form, the main things to be studied—the key factors, concepts, or variables—and the presumed relationships among them” (40).

#### 2- Social determinants of health (SDH)

There is no clear and single definition of the social determinants of health, but “Social determinants of health refer to both specific features of and pathways by which societal conditions affect health and that potentially can be altered by informed action” (41).

United States Centers for Disease Control states another definition of social determinants of health as “life-enhancing resources, such as food supply, housing, economic and social relationships, transportation, education, and health care, whose distribution across populations effectively determines length and quality of life” (42).

Based on WHO definition “The social determinants of health (SDH) are the conditions, in which people are born, grow, work, live, and age and the wider set of forces and systems shaping the conditions of daily life.” (11). “The complex, integrated, and overlapping social structures and economic systems that are responsible for most health inequities included the social environment, physical environment, health services, and structural and societal factors” (43).

### Search Strategy and search engines

This was a narrative study to presents conceptual models of SDH that performed to review publications in English language before Mar 2015 and the PubMed, Scopus, Emerald, Elsevier, Ovid, Google Scholar, Springer, ProQuest, WHO and Word Bank databases were used. Participants, interventions, comparisons and outcomes (PICO) strategy along with following keywords obtained from PubMed (MeSH terms) were used for searching: socioeconomic status, factor/s, position, characteristics, determinants, stratification, social determinants, social class/es, social condition, social stratification, living conditions, standard of living, living standard/s AND conceptual OR theoretical framework OR model.

### Exclusion and inclusion criteria

All publications such as reviews, systematic reviews, and meta-analysis, qualitative, quantitative, books, reports and thesis were accepted if they had been published in English language and full text was accessible.

### Study selection

The titles of the retrieved citations checked independently by two reviewers according to the above selection criteria. Full-text copies of potentially relevant studies obtained and their appropriateness for inclusion independently assessed by two reviewers. Literature that does not fulfill all of the inclusion criteria was excluded.

### Critical appraisal

Quality assessments of publications independently were carried out on each study by two reviewers using the relevant version of the Critical Appraisal Skills Programme (CASP) for qualitative research. Similarly, the Preferred Reporting Items for Systematic Reviews and Meta-Analyses (PRISMA) checklist used for assessing systematic reviews.

### Analysis, data synthesis, and reporting

The PRISMA checklist was used to guide the reporting of the systematic review.

## Results

### Descriptive findings

In all, 248 publications were retrieved. 154 citations were excluded because of irrelevant (n=139), duplicates (n=10), and full text was not available (n=5). After studying the 94 remaining publications, 21 citations were extracted and presented.

### Conceptual models

Twenty-one extracted conceptual models of SDH divided as follow by historical perspective:

#### 1- Models before year 1995

This era included 4 models such as Williams’ conceptual framework, the social production of disease, rainbow- like layers framework, and selection and causation model (44–47).

#### 2- Models between 1995 and 2005

This era included 13 models:

The Bullseye model of social determinants of health, the social and economic determinants of health model, MacArthur research network on SES and health model, multiple life-course influences model, determinants of health- sector outcomes conceptual framework, a multivariate, casual and life course framework, socioeconomic determinants of health framework, World Bank’s poverty reduction strategy papers(PRSP) pathways framework, PROGRESS-Plus model, a public health model of the social determinants of health, Alberta social determinants of health framework, social determinants of health causal pathway model and the conceptual model of health status predictors(34, 48–58).

#### 3- Models beyond 2005

This era included 4 models such as the conceptual framework for action on the social determinants of health (WHO approach), NICE’s emerging conceptual framework for public health, County health rankings model, Australian institute of health and welfare (AIHW) (59–63).

### SES indicators

Extracted conceptual models revealed that various variables can be considered as SES indicators of health in household level survey. We divided these indicators into 3 categories:

#### 1- Common or classic indicators

Included education, income, and occupation: Education is the most basic and widely used indicator of socioeconomic status (64) that has a complicated mechanism and relationship with two key variables: income and occupation (65). Usually, educated people have better knowledge about risky health behaviors, relevant healthcare and use health care services effectively (66, 67). People with high education levels are more likely to be socialized in health promoting behaviors, lifestyles and have a better job and economic situation (68, 69). In general, education is a determinant of future income and job statute (70, 71).

Income is the second major variable in determining the socioeconomic conditions. Income has a significant relation with employment, work condition, and reflection of the resources available in a given time (72). Income represents the flow of economic resources in a period of time (64).

Occupation: or employment represents individual’s position in social structure and explains access to resources, expose to psychological and physical risk factors and impact on lifestyle (64).

Income, education, and occupation- three main classical indicators (16) - are the key variables in determining SES (73) and represents individuals position in social hierarchy (16). In the epidemiological literature, these variables are considered as the traditional measures of SES (64).

#### 2- Fixed and demographic indicators

Included genetics, sex/gender, ethnicity/race, age, BMI, marital status, and religion:

Genetics: in addition to age and gender, is a factor affecting health changed or modified (74).

Sex/Gender: at the end of 20^th^ century, life expectancy was higher in women than men. However, this difference has been attributed to biological differences; but, it is important to study interaction between these differences and physical and social environments (75). Men and women’s social and economic roles have a significant effect on their health risk factors (76).

Ethnicity/race: traditions, cultural and religious beliefs, health behaviors and healthcare services consumption have a close relationship with race or ethnicity (77).

Age (life course): refer to health status in a given age and not only included current situation, but also considers the fatal, early life condition and later on (78).

BMI: is calculated by weight in kilograms divided by the square of height in meters (79). In some conceptual models, this variable is mentioned as an index that reflects lifestyle risk factors (50) and used as an objective indicator of socioeconomic at micro level (53).

Marital status: married people are healthier than single ones. In addition, marital status has a relation with income and education; hence, if there is enough data available, it can be considered as a variable by researchers (75).

Religion: researches did not pay enough attention to religion and religious affiliation and identity as a dimension of social identity and a factor that involved in socioeconomic inequalities in health. While, in contemporary societies, religious identity and particularly religious minorities reflect their social status on one hand and social resources provided to them on the other hand (80).

#### 3- Proxy and complementary indicators

Included wealth and asset, household size, social capital and trust, social and family support, utilization and access to healthcare services, health behavior, housing, culture or cultural factors, place of residence and social and family safety.

Wealth and asset is a proxy of income and reflects the assets gathered during people’s life (81) and can be a complementary measure for SES (82). Income and wealth have a positive relation with themselves (83). Wealth, in addition to income, includes financial and physical property such as house, car, investments, heritage and pension (84).

Household size is a complementary indicator beside income, housing, wealth and asset (84). Crowding index can be calculated from household size, as a proportion of the number of people who live in the dwelling, per number of rooms in the place of residence, except kitchen and bathroom (83).

Social capital and trust refer to current level of community social trust, how much people help each other based on self or collective interests and the degree of involvement in social issues (85).

Social and family support is a component of the social environment that is more important than the physical environment. Social isolation and non-participation in social networks are considered as strong predictors of health. Social networks and social cohesion are affected by larger social environment (15).

Utilization and access to health care services: that will vary based on the socioeconomic conditions (15).

Health behavior: behavioral and lifestyle factors are the reason for more than half of premature death and can be varied according to SES (15). Regular physical activity, healthy diet and nutrition, non-smoking, no drug and alcohol abuse, healthy sexual behavior are some part of healthy lifestyle and behavior’s factors.

Housing: features such as ownership and facilities evaluate financial aspects of SES and the main indicator of people’s property. Therefore, housing is considered as an important, multifaceted and very difficult indicator to interpret the SES (86).

Culture or cultural factors refers to a set of implicit guides that people as a member of particular group or community inherit it and determines the worldview, emotional experiences as well as behavior in communication with others, the supernatural and the natural environment (87). In general, this factor refers to the behavioral patterns and norms accepted by certain groups (75).

Place of residence: refers to differences between urban and rural, regional groups, capital vs. other region and another part of cities (different and crowded regions and districts) (88).

Social and family safety reflects the violence in neighborhoods and houses as well as injuries caused by unintentional events. Most injuries are predictable and preventable (89).

## Discussion

### Conceptual models

The Williams’ conceptual framework was oldest model while the Australian Institute of Health and Welfare (AIHW) was newest ones. Williams’s model explains the relation between the SES, socio-psychological factors and medical care with health outcomes (44).

The AIHW model argued that people’s health and well-being arises from complex interactions among biological, lifestyle, socioeconomic, environmental and social factors which many of them can be modified with medical care and other interventions. In this model, factors affecting health are divided into 4 groups: 1) broad features of society and environmental factors, 2) socioeconomic characteristics, awareness, attitude and health belief, 3) health behavior and psychological and safety factors, 4) biological factors. In the model, the first and basic factors (features of society and environmental factors) determine the nature of next group (socioeconomic characteristics), while, these two groups from the health behavior of people and effect on psychological and safety factors. Finally, these three groups can effect on biological factors that have health effects in different ways. In whole process, various mentioned factors interact with genetic composition. Besides, the factors listed within the major factors often interact closely with each other. Occupation, education, financial resources, social support networks and social position of people can effect on their health and can also increase the health inequalities in community (63).

Among models presented, only three models (Selection and Causation model, the Bullseye Model of Social Determinants of Health and Conceptual Model of Health Status Predictors) studied the life course approach. The evaluations of life course are an innovative and complementary way for studying other indicators of socioeconomic conditions in healthcare system (90). It provides a significant opportunity to explain causality ways of socioeconomic factors of health. In general, life course approach represents a useful framework for describing and understanding social patterns of diseases at individual and collective level (84).

The Bullseye model aims were to understand the health determinants with life course approach. In this model, life course (from birth to death) is divided into 3 levels. Higher levels of the socioeconomic and psychological determinants of health are related to socioeconomic features of a nation. Factors related to civil society are located in intermediate level related to features of social organization such as institutional responsibility, social trust, social cohesion and access to social goods (like health care and education). Finally, intimate family territory and environment are located in micro level which includes economic condition and individual support network (91). This model represents the relationship between these three levels as a circle and communities are represented by three concentric circles and each circle includes determinants mentioned earlier. In addition, the central role of life is shown as an arrow which passes through layers of three circles. Population health is an emergent function of consistent opportunities and vulnerability of people in the population resulted from interactions among financial, behavioral, cognitive and emotional factors in life as well as psychological and SES of people in family, civil and national level. Therefore, psychological and SES change the people conditions and have different effects on their view about health. People and groups can change society by their response to environmental factors in which they are growing up, living and working (48, 91).

A conceptual model of predictive factors of health status was presented that shows a phased approach to life. In this model, various socioeconomic indicators with a focus on education, occupation, and income were described as health determinants. Self-report predictive indicators were divided into 4 categories: a) historical SEIs, b) lifestyle risk factors, c) current SEIs, d) disease indicators (58).

Mackenbach model or selection and causation model explain the socioeconomic inequalities of health based on life course approach. In the model, effects of socioeconomic factors in both selection and causation from childhood to adulthood are represented like a chain. Meanwhile, childhood environment, cultural factors, and biological-psychological factors are also affecting the health determinants through both selection and causation (47, 59, 92, 93).

Rainbow- like layers framework is cited in many articles and reports (59, 92, 94–104). The model presented in 1999 looks like a rainbow. The authors believe that 5 different layers should be considered for understanding the health determinants.

WHO model compared with other models have a systematic integrated and dynamic approach. The model with systematic integrated and dynamic approach not only considers biological components and the casual and interactive way but also it contains other model’s factors (multiple levels, main and intermediary factors and effective factors at different times). The model explicitly considers the relationship between agents, non-linear correlation, and feedback (105). In this model, there are two categories of social determinants: A) structural determinants: two main components; a.1) socioeconomic and political context: consists of components of governance, macroeconomic and social policy, public policy, social culture, and values; a.2) socioeconomic position: such as education, occupation, income, social class, ethnicity/race and gender. These two groups have a reciprocal interaction and can be affected by each other. B) Intermediary determinants: including environmental condition, socio-psychological factors, behavioral factors, biological factors and health system. In this model, the relation between intermediary determinants and structural determinants is created by social cohesion or social capital mentioned as crosscutting determinant (43, 59–60) and the structural factors affect the health and welfare through affecting on intermediate factors (106). In recent years, this model has been developed and localized in some countries, including Iran (107).

The overall comparison of different models before year 1995 such as Williams’ conceptual framework, the social production of disease, rainbow- like layers framework, and selection and causation model were focused on identifying determinants of health and the relationship between them. Models between 1995 and 2005 have been focused on the classification of determinants and their relationship in the form of general and universal models. Finally, Models beyond 2005 have more emphasis on the role of SES and have focused on reducing health inequalities through improving the SES.

### SES indicators

In this study, 20 indicators were referred as socioeconomic or social determinants. To have a better understanding of health differences and inequalities, we should use the different SES indicators in health researches (84). Although there is no best indicator (86, 108) adopted with all goals of the study and applied in all times and places (86), but income, education and occupation were mentioned as the main SES indicators in all scientific documents and conceptual models (16, 44–64, 73).

In addition, to the key SES indicators and demographic indicators such as age, sex/gender, marital status, genetics, ethnicity, religion, household size and BMI, other main indicators such as housing, place of residence, wealth and assets, health behaviors, access and use of healthcare services and social capital (social and family support and safety and cultural context) were also referred in these models considered by health policymakers to study socioeconomic inequalities for the purpose of policy interventions. Health inequalities were conducted in developed countries (84) and studied indicators and models are not suitable for developing countries. Hence, these countries need to study, design and define the models and socioeconomic determinants in accordance with their socioeconomic conditions.

### Limitation of the study

In this study, we could not access to ISI Web of Science database in the search period and the lack of required framework for comparative study and systematic registration of socioeconomic indicators was the other limitation of the present study.

## Conclusion

The present study has two clear results. First, reduction or elimination of socioeconomic inequalities requires the understanding of the variables, mechanism and pathway causality. This understanding can be obtained by existing conceptual models. Since these models and their indicators are designed for developed countries and there is little consistency with indicators and conditions of developing countries; lack of a specific model for developing and Islamic countries is clear. Second, there is no gold standard related to SES indicators. Therefore, we recommend the study of multiple indicators with life course approach.

## Ethical considerations

Ethical issues (Including plagiarism, informed consent, misconduct, data fabrication and/or falsification, double publication and/or submission, redundancy, etc.) have been completely observed by the authors.
